# Adaptive Neuroplasticity in Brain Injury Recovery: Strategies and Insights

**DOI:** 10.7759/cureus.45873

**Published:** 2023-09-24

**Authors:** Vaishnavi Zotey, Amol Andhale, Tejas Shegekar, Anup Juganavar

**Affiliations:** 1 Medicine, Jawaharlal Nehru Medical College, Datta Meghe Institute of Higher Education and Research, Wardha, IND

**Keywords:** personalized neurorehabilitation, sensory recovery, virtual reality-based rehabilitation, brain-computer interfaces, motor skill regeneration, cognitive rehabilitation, neural recovery, rehabilitation strategies, brain injury, neuroplasticity

## Abstract

This review addresses the relationship between neuroplasticity and recovery from brain damage. Neuroplasticity's ability to adapt becomes crucial since brain injuries frequently result in severe impairments. We begin by describing the fundamentals of neuroplasticity and how it relates to rehabilitation. Examining different forms of brain injuries and their neurological effects highlights the complex difficulties in rehabilitation. By revealing cellular processes, we shed light on synaptic adaptability following damage. Our study of synaptic plasticity digs into axonal sprouting, dendritic remodeling, and the balance of long-term potentiation. These processes depict neural resilience amid change. Then, after damage, we investigate immediate and slow neuroplastic alterations, separating reorganizations that are adaptive from those that are maladaptive. As we go on to rehabilitation, we evaluate techniques that use neuroplasticity's potential. These methods take advantage of the brain's plasticity for healing, from virtual reality and brain-computer interfaces to constraint-induced movement therapy. Ethics and individualized neurorehabilitation are explored. We scrutinize the promise of combination therapy and the difficulties in putting new knowledge into clinical practice. In conclusion, this analysis highlights neuroplasticity's critical role in brain injury recovery, providing sophisticated approaches to improve life after damage.

## Introduction and background

The extraordinary capacity of the brain to modify its structure and function in response to environmental and experience changes is known as neuroplasticity, sometimes called brain plasticity [1]. The term *synaptic plasticity* refers to changes in the strength and effectiveness of connections between neurons, whereas *structural plasticity* refers to the development of new brain pathways and synapses [2]. Neuroplasticity plays a crucial role in determining recovery outcomes in brain injury rehabilitation. The brain's inherent ability to reconstruct itself after brain injury, whether brought on by trauma or acquired disorders, becomes an essential consideration in rehabilitation. For those trying to restore lost abilities, accommodate disabilities, and eventually improve their general quality of life, this reorganization potential holds hope [3]. There are many different types of brain injuries, including traumatic brain injuries (TBI) brought on by external factors like accidents or falls and acquired brain injuries brought on by occurrences like strokes, tumors, or infections. These wounds can significantly impact neuronal networks, causing connections between different brain areas to disrupt and resulting in functional deficiencies [4]. Brain injuries can have other effects depending on where, how severe, and what kind of damage they are. Notably, the brain's propensity for neuroplasticity allows it to make new connections and reroute neuronal pathways to partially make up for these disturbances, frequently in response to extensive rehabilitation efforts [5].

## Review

Methodology

**Figure 1:**
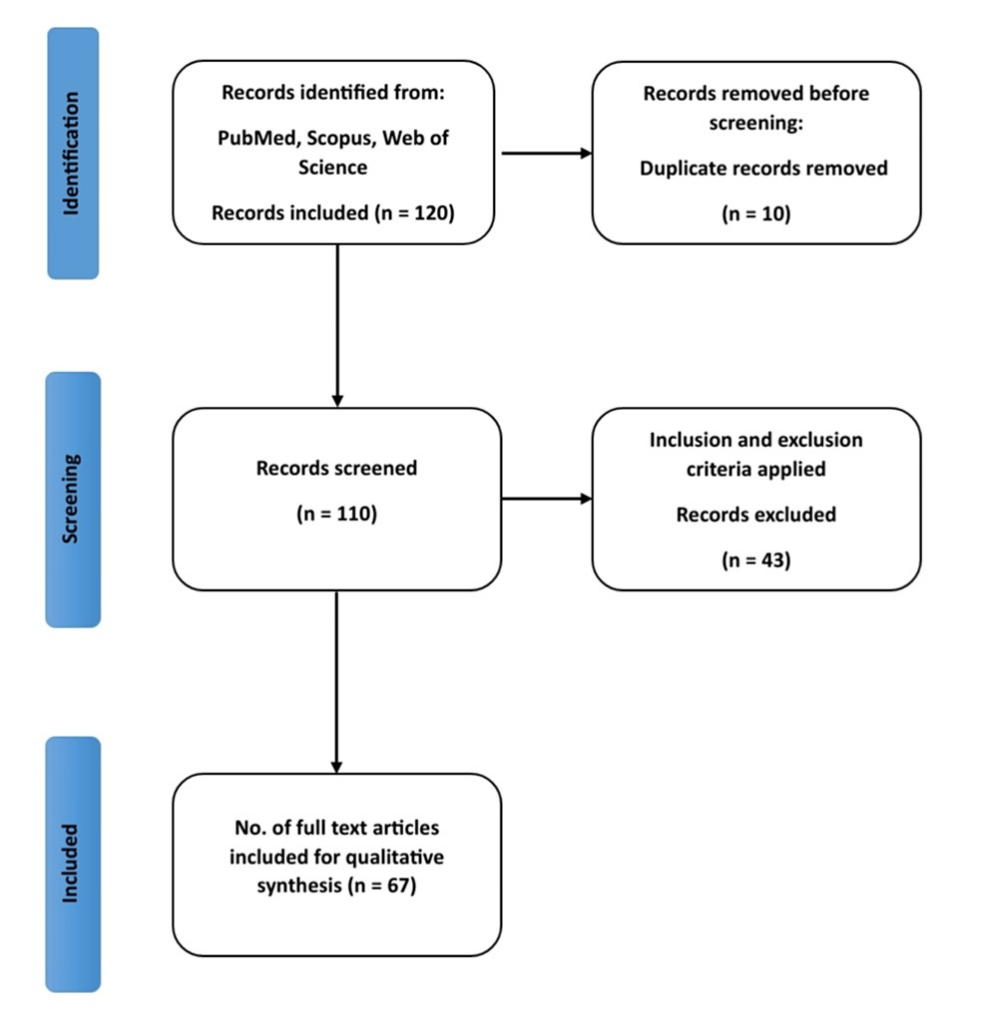
The PRISMA flow diagram illustrates the process of study selection. PRISMA, Preferred Reporting Items for Systematic Reviews and Meta-Analyses

In this systematic review, a thorough search strategy was meticulously executed to identify pertinent articles from well-established databases. The search encompassed prominent platforms such as PubMed, Web of Science, and Scopus. We considered articles published between January 2000 and September 2022 to ensure a contemporary and comprehensive review. To cast a wide net, a set of key terms and MeSH terms was employed, including "Neuroplasticity," "Brain Injury," "Rehabilitation," "Neurorehabilitation". In adherence to rigorous inclusion criteria, we prioritized studies that specifically addressed neuroplasticity mechanisms and rehabilitation strategies following brain injury. Our focus was on human studies, encompassing randomized controlled trials, systematic reviews, meta-analyses, and comprehensive investigations exploring both immediate and delayed neuroplastic changes, as well as rehabilitation interventions capitalizing on neuroplasticity and their subsequent outcomes. Exclusion criteria were applied to studies that diverged from these parameters, including those conducted on animals, published in non-English languages, or lacking relevance. After an exhaustive screening process involving initial database searches, 120 articles were identified. Subsequent evaluations based on titles and abstracts led to the selection 110 articles for a thorough full-text review. Ultimately, 67 articles met the stringent inclusion criteria and were thus incorporated into the final review. For visual clarity, a Preferred Reporting Items for Systematic Reviews and Meta-Analyses (PRISMA) flow diagram (Figure 1) has been provided to illustrate the stepwise article selection process.

Neuroplasticity: mechanisms and adaptations

Cellular and Molecular Mechanisms of Neuroplasticity

The brain's unique capacity for reorganization and adaptation on intricate cellular and molecular pathways is known as neuroplasticity. Long-term depression (LTD) and long-term potentiation (LTP) are crucial processes at the cellular level. In contrast to LTD, which weakens synapses by lowering receptor sensitivity, LTP increases neurotransmitter release and synaptic strength [6]. Neuroplasticity depends on complex signaling networks at the molecular level. Calcium ions control synaptic alterations, second messengers such as cyclic adenosine monophosphate (cAMP), and protein kinases [7]. Circuit rewiring is facilitated by structural plasticity, which includes dendritic branching and axonal sprouting [8].

Structural Plasticity: Dendritic Remodeling and Axonal Sprouting

A key aspect of neuroplasticity, called structural plasticity, includes dynamic alterations in neuronal architecture, such as dendritic remodeling and axonal sprouting. Dendritic remodeling modifies dendritic length, branching patterns, and spine density, promoting new synapses' growth and reinforcing existing connections [9]. On the other hand, axonal sprouting refers to the expansion of new axonal branches from preexisting neurons. This process frequently happens in reaction to an injury or functional requirements, allowing the creation of new connections and pathways. By rearranging neuronal networks around injured regions, axonal sprouting can aid in active recovery following brain trauma [10].

Synaptic Plasticity: LTP and LTD

The capacity of synapses to experience long-lasting changes in strength based on brain activity is known as synaptic plasticity, a key component of neuroplasticity. LTP and LTD are two common types of synaptic plasticity. Because pre- and postsynaptic neurons fire repeatedly and in sync, LTP symbolizes the ongoing strengthening of synaptic connections [6]. Contrarily, LTD causes synaptic connections to continue to deteriorate, frequently as a result of low-frequency stimulation or asynchronous firing [11].

Neuroplasticity in response to brain injury

Immediate Neuroplastic Changes

Rapid adaptation mechanisms that happen soon after a brain injury are called immediate neuroplastic alterations. Cellular alterations and synaptic plasticity are frequently involved in these changes. For instance, within hours to days following TBI, investigations have shown differences in synapse strength and neurotransmitter release at impacted neuronal circuits [12].

Delayed Neuroplastic Changes

The brain may continue to adapt and reorganize for a considerable time after damage, as seen by delayed neuroplastic alterations. This phenomenon can include structural alterations such as axonal sprouting and dendritic remodeling. According to research results, injured areas significantly increase their axonal branching and collateral creation weeks to months after injury, which aids in functional recovery and compensation [13].

Compensation vs. Maladaptation: How Brain Reorganization Affects Function

In recovering from brain damage, neuroplasticity contributes to both compensatory behavior and possible functional maladaptation. To retain or restore function after brain damage, compensating includes the brain shifting activities to unaffected areas. This adaptive reaction frequently results in enhanced performance in previously degraded tasks. Maladaptation, however, can happen when compensatory processes unintentionally prevent optimum healing. Overreliance on other routes may prevent the activation of initial pathways, hence restricting progress in general. Effective rehabilitation requires balancing compensating for deficits and re-establishing healthy brain connections [14].

Rehabilitation strategies exploiting neuroplasticity

Constraint-Induced Movement Therapy and Repetitive Task Training

Utilizing neuroplasticity in rehabilitation techniques aims to maximize the brain's ability for reorganization to speed up healing following brain damage. Limiting the unaffected leg forces patients to utilize the afflicted limb for daily tasks as part of constraint-induced movement therapy (CIMT). This rigorous training promotes brain reorganization and improved function [15]. The goal of repetitive task training (RTT) is to practice particular motor functions repeatedly and consistently. This method facilitates the reacquisition of motor skills by encouraging synapse strengthening and cortical reorganization [16]. Both CIMT and RTT emphasize the idea of experience-dependent neuroplasticity and show promise for enhancing the results of rehabilitation.

Cognitive Rehabilitation and Brain-Computer Interfaces

To restore cognitive function after brain damage, cognitive rehabilitation techniques make use of neuroplasticity. Through exercises and activities that are organized and that promote brain reorganization, these therapies focus on attention, memory, and executive functions [17]. Brain-computer interfaces (BCIs) are a cutting-edge method of neurorehabilitation. BCIs provide direct brain-to-external device connection, allowing people with motor disabilities to operate computers or prosthetics using their neural signals [18]. BCIs and cognitive rehabilitation are two examples of how technology and neuroplasticity might be used to speed post-injury healing.

Virtual Reality and Gamification for Motor and Cognitive Recovery

Gamification and virtual reality (VR) have become dynamic tools in neurorehabilitation that use neuroplasticity to encourage motor and cognitive recovery. With the help of immersive worlds created by VR, patients may participate in interactive activities that boost motivation and facilitate neural reorganization [19]. VR-based exercises for motor rehabilitation provide task-specific training, allowing patients to practice motions in a safe but entertaining environment. Real-time feedback enables users to modify and improve their actions, fostering skill development and relearning [20]. To exercise several cognitive domains, such as attention, memory, problem-solving, and decision-making, VR and gamification provide problems. These exercises take advantage of the brain's capacity for adaptation to enhance cognitive performance through regular participation and variable difficulties [21]. These technological advancements provide individualized experiences that adapt to each person's growth and ability. In addition, VR and gamification's entertaining and engaging aspects improve patient compliance and stimulate regular engagement, which is essential for neuroplastic changes to occur [22].

Neurorehabilitation techniques

Physical Therapy and Motor Skill Regeneration

To restore motor function and encourage the regeneration of motor skills after brain damage, physical therapy is a cornerstone of neurorehabilitation. Physical therapy methods use neuroplasticity to tap into the brain's adaptability and capability for reorganization. Task-specific training entails engaging in practical drills that mirror actual work. Exercises that are done repeatedly and intensely help to develop new brain pathways and improve motor abilities. This strategy uses the brain's capacity to change its wiring due to experience [23]. The intact limb is restrained with CIMT, which forces patients to use the injured limb for daily tasks. This rigorous exercise promotes neuronal rewiring and improves motor performance by making the brain devote resources to the damaged limb [15]. Functional electrical stimulation (FES) involves stimulating weak muscles with electrical currents. This method causes muscular contractions and encourages neuroplastic modifications in the peripheral and central nervous systems, improving motor control [24]. Robotic devices support patients with repeated motions in robot-assisted therapy at varying degrees. These tools provide fine control over movement patterns, allowing motor relearning and encouraging brain plasticity [25].

Speech and Language Therapy in Brain Injury Recovery

Speech and language therapy is crucial to neurorehabilitation because it aims to recover linguistic and communicative skills after brain damage. Speech and language therapies use neuroplasticity to help the brain's adaptable ability to reorganize and recover.

Aphasia, a linguistic problem frequently brought on by brain damage, is treated using specialized methods called aphasia therapy. These include melodic intonation treatment, constraint-induced language therapy, and semantic feature analysis, which all use neuroplasticity to retrain language centers and encourage functional communication [26]. Augmentative and alternative communication (AAC) methods are utilized with those with significant language impairments. These techniques, including message boards or electronic gadgets, enable patients to interact through alternate channels while encouraging brain reorganization related to language processing [27]. The capacity to comprehend and make use of nonliteral language, gestures, and social cues can be impacted by brain damage. Through systematic interventions that use real-world situations to promote adaptive neuroplastic changes, pragmatic language training focuses on enhancing these elements [17].

Occupational Therapy for Functional Independence

A key component of neurorehabilitation, occupational therapy focuses on repairing and increasing patients' capacities to carry out meaningful tasks and attain functional independence following brain damage. Interventions in occupational therapy focus on particular abilities and mental processes needed for daily living by taking advantage of neuroplasticity.

Occupational therapists use cognitive training activities in cognitive rehabilitation to improve patients' memory, attention, problem-solving, and decision-making abilities. These exercises encourage neuroplastic adjustments that enhance cognitive function and enable people to perform necessary tasks. Training in activities of daily living (ADL), such as dressing, bathing, cooking, and grooming, is emphasized in occupational therapy. These exercises use a variety of cognitive and motor processes, promoting functional recovery and relearning [17].

Neuroplasticity and sensory recovery

Sensory-Motor Integration and Sensory Relearning

In the study of neurorehabilitation, it is essential to comprehend how neuroplasticity affects sensory recovery, particularly in relation to sensory-motor integration and sensory relearning. The brain's incredible capacity to change its structure and function in response to sensory impairments or injury is known as neuroplasticity. Neuroplasticity enables the brain to adjust to changes in sensory input, which is important for tasks like coordinated movement and sensory-motor integration. This adaptability can be utilized through therapeutic strategies that support sensory recovery.

One prominent illustration of a therapy approach that uses neuroplasticity is sensory relearning. Training in sensory discrimination has been found to be helpful in stroke patients, according to research by Carey et al. This training aimed to improve motor coordination and sensory perception in those with poor sensory-motor integration. The findings demonstrated improvements in motor and sensory recovery, demonstrating the potential of neuroplasticity-driven therapies to promote sensory healing in patients with neurological disorders [28].

Auditory Rehabilitation Approaches

Auditory rehabilitation techniques use neuroplasticity to improve speech and hearing comprehension in people with hearing loss. Cochlear implants and audio training programs are frequent therapies in this situation. The effect of a sensitive period on the development of the central auditory system in children with cochlear implants was studied. The study's findings, which highlight the significance of neuroplasticity in auditory sensory recovery, suggest that early cochlear implant treatments may improve auditory and speech development [29]. These illustrations show how neuroplasticity-based therapies, in both the sensory-motor and sensory perception domains, can support sensory rehabilitation and raise living standards for people with sensory impairments.

Neuroplasticity in cognitive rehabilitation

Memory Training and Cognitive Remediation

Cognitive rehabilitation techniques use the brain's incredible capacity for self-organization, or neuroplasticity, to enhance cognitive function following brain damage. This is especially true of memory training and cognitive rehabilitation methods, which use focused treatments to improve memory and general cognitive abilities.

A technique for memory training called the spaced retrieval rechnique makes use of the brain's capacity to organize and store knowledge through repeated recall. It entails gradually extending the spaces between distinctly remembered facts. For example, a patient may remember a certain phrase or fact right away after hearing it, then a few seconds later, then a minute later, and so on. The brain's capacity to recover information is improved with time by strengthening memory consolidation, neuronal connections and gradually expanding recall intervals [30]. Programs for computer-based cognitive training are another kind of cognitive rehabilitation that makes use of neuroplasticity. These activities are usually entertaining and challenging to test different cognitive abilities, including executive function, memory, attention, and memory recall. Individuals are exposed to mental problems repeatedly while they participate in these workouts. Individuals gradually increase their performance in these activities due to reinforced neural networks and improved cognitive processes, demonstrating the brain's capacity to adapt and remodel itself [31].

Attention Enhancement Strategies

Neuroplasticity is used by attention-enhancement techniques in cognitive rehabilitation to aid cognitive recovery, particularly in the area of attention. A strategy called attention process training uses methodical exercises to improve the regulation of attentional functions. Patients may encourage their brain to adapt and reorganize the neural circuits in charge of attention by frequently doing activities that call for different types of attention, such as sustained concentration, selective attention, and split attention. Due to the recurrent nature of these activities, neuroplastic changes are triggered, which gradually increase attentional capacities [17]. The brain's potential to adapt and regain cognitive skills is demonstrated by cognitive rehabilitation methods that use neuroplasticity, such as memory training, cognitive remediation, and attention enhancement approaches. These methods not only enhance cognitive recovery following brain damage but also highlight how adaptive and dynamic the human brain is.

Technological Advances in Neuroplasticity-Based Rehabilitation

By creating immersive and engaging settings, VR and augmented reality (AR) technologies have revolutionized neurorehabilitation. Patients can participate in various activities and tasks related to their recovery goals in these technologically created simulated environments. Interventions using VR and AR take advantage of neuroplasticity by stimulating the brain with exciting experiences. Within these virtual settings, patients can practice their physical skills, cognitive activities, and even social interactions. The brain rewires neural connections in response to these events, promoting learning and functional recovery [19]. BCIs allow the brain and outside technologies to communicate directly. These interfaces translate cerebral activity into usable instructions that operate machinery like computers, prosthetic limbs, or assistive technology. BCIs use neuroplasticity by enabling the brain to change and learn how to produce certain neural signals that result in desired behaviors. By activating brain networks related to motor control and intention, this approach enhances functional recovery [18]. Robotic equipment is used in robot-assisted therapy to aid patients with mobility drills. These tools offer repeated, regulated motions that let patients practice certain motor functions. Robotic treatments encourage motor relearning, which takes advantage of neuroplasticity. The brain adjusts to the robotic supervision, enhancing movement patterns and honing motor abilities. This procedure starts a brain reorganization process that enhances motor control [25].

Technological advancements, notably noninvasive brain stimulation techniques like transcranial magnetic stimulation (TMS) and transcranial direct current stimulation (tDCS) have considerably broadened the scope and effectiveness of neuroplasticity-based therapy in recent years. TMS, a method that uses magnetic fields to stimulate brain activity, has become popular because it can alter neuronal circuits and encourage functional recovery in a variety of neurological diseases [32]. However, tDCS, which involves passing a small electrical current across the scalp, has demonstrated promise in promoting neuroplasticity and increasing motor and cognitive abilities [33]. These noninvasive brain stimulation techniques provide focused treatments that adjust to specific patient demands and brain responses, optimizing the rehabilitation process. They also offer a personalized approach to neurorehabilitation.

Real-time functional magnetic resonance imaging (rt-fMRI) and neurofeedback allow people to monitor and control their brain activity. These tools enable users to learn how to self-regulate their neurological processes by giving feedback on particular brain states or patterns. Neurofeedback and rt-fMRI therapies leverage neuroplasticity by using the brain's ability to adapt and remodel itself. Patients gain the ability to control their brain activity, which encourages self-regulation and cognitive development [34].

Personalized approaches to neurorehabilitation

Tailoring Rehabilitation Programs to Individual Patient Profiles

Recognizing the individuality of each patient's brain and damage has prompted the creation of tailored strategies in the field of neurorehabilitation that take into account distinct patient profiles. This customized strategy maximizes the efficacy of rehabilitation therapies by treating individual impairments, utilizing neuroplasticity, and coordinating with the patient's skills and goals.

Cognitive profiles for the purpose of creating individualized therapies and evaluating a patient's cognitive strengths and limitations are essential. For instance, the rehabilitation program can concentrate on memory training activities if a patient exhibits memory problems after suffering a brain injury. These exercises test and stimulate the brain's ability to undergo neuroplastic changes in the particular cognitive area that needs strengthening [35].

Adopting personalized methods of neurorehabilitation has become a viable path in the field of motor functioning. These customized approaches consider every patient's distinct demands and recovery trajectories. Personalization requires taking into account the patient's age, concomitant conditions, cognitive ability, and the severity and kind of motor impairments. Functional magnetic resonance imaging (fMRI) has allowed for more excellent knowledge of how each person's brain reacts to rehabilitation interventions, allowing for the fine-tuning of therapies to maximize neuroplasticity [36]. Furthermore, wearable technology and sensor-based evaluations provide real-time monitoring of motor function, assisting in the adjusting of rehabilitation protocols in accordance with a patient's development [37]. Such individualized strategies improve motor recovery results and patients' overall quality of life during neurorehabilitation.

Neurorehabilitation, specifically tailored to each patient, goes beyond physical healing. It considers psychological elements, including levels of motivation, societal networks of support, and emotional health. By addressing these issues, the rehabilitation program improves patient participation, cultivates a positive outlook, and generates an atmosphere that supports neuroplastic improvements [2].

Biomarkers for Predicting Neuroplasticity Response

As a result of advancements in neuroscience, it is now possible to utilize biomarkers to anticipate how a person's brain will respond to neurorehabilitation, potentially allowing for the customization of treatment plans based on expected neuroplasticity. Clinicians may map and visualize brain activity using fMRI and diffusion tensor imaging (DTI). These biomarkers give information on the areas of the brain that are most receptive to neuroplastic changes, assisting in selecting suitable therapies and monitoring development over time [38]. Genetic variables influence the propensity for neuroplasticity in an individual. Clinicians can forecast which patients would benefit from particular therapies by finding genetic markers linked to neuroplastic response. This enables a more focused and unique recovery strategy [2]. Event-related potentials (ERPs), a type of electrophysiological marker, shed light on the neuronal excitability of the brain and how it reacts to treatments. Monitoring these indicators can aid in directing the modification of rehabilitation procedures and ensuring that medicines successfully foster neuroplastic changes [39].

A comparative overview of various neuroplasticity-based rehabilitation strategies is mentioned in Table 1 [15,17,19,40-56].

**Table 1:** Comparative overview of neuroplasticity-based rehabilitation strategies. Sources: [15,17,19,40–56].

Rehabilitation strategy	Mechanism of action	Targeted outcome	Key benefits
Constraint-induced movement therapy	Forced use of impaired limbs for functional tasks	Motor recovery and skill improvement	Promotes neuroplasticity through intense use of affected limbs
Virtual reality and gamification	Immersive environments for motor and cognitive tasks	Motor and cognitive improvement	Explores virtual neural pathways, enhances motivation
Brain-computer interfaces	Brain signals control external devices	Motor and communication recovery	Directly engages neuroplastic responses in control regions
Cognitive rehabilitation	Cognitive exercises to improve neural function	Cognitive enhancement	Promotes synaptic strengthening in cognitive circuits
Robot-assisted therapy	Robotic devices guide controlled movements	Motor skill improvement	Facilitates neural relearning and sensorimotor integration
Transcranial magnetic stimulation	Noninvasive brain stimulation	Motor and cognitive recovery	Induces neuroplastic changes through modulating neural activity
Personalized neurorehabilitation	Tailored programs based on patient profiles	Enhanced recovery outcomes	Addresses individual needs and optimizes neuroplasticity
Multimodal Rehabilitation	Integration of multiple therapies	Holistic recovery	Harnesses synergistic neuroplastic effects
Sensorimotor Integration Training	Integrating sensory and motor exercises	Improved sensory-motor coordination	Facilitates neural connections between sensory and motor regions
Hybrid virtual reality-based therapy	Merging virtual reality with physical exercises	Motor and cognitive recovery	Provides engaging, immersive rehabilitation with neuroplastic benefits
Combination of brain stimulation with rehabilitation	Pairing brain stimulation with motor training	Enhanced motor and cognitive recovery	Amplifies neuroplastic responses and recovery progress

Challenges and future directions

Ethical Considerations in Neurorehabilitation

The importance of ethical issues is growing as neurorehabilitation technology develops. The use of novel technologies and therapies that take advantage of neuroplasticity raises a number of ethical quandaries. Informed consent is crucial to ensure that patients thoroughly comprehend the possible advantages, dangers, and uncertainties of neurorehabilitation therapies. Obtaining thorough, informed permission can be difficult due to the intricacy of some interventions, especially those employing innovative technology [57]. BCIs and VR systems gather private behavioral and neurological data. Ethical problems include preserving patient privacy, guaranteeing data security, and gaining informed permission for data use [58].

Achieving equality in neurorehabilitation is essential to preventing the escalation of already-existing health disparities, especially for economically disadvantaged subjects. Vulnerable groups are disproportionately impacted by health inequities, particularly in the setting of neurorehabilitation [59]. To ensure that underprivileged people receive the treatment they require, it is crucial to remove access hurdles such as financial limitations, transportation problems, and a lack of service availability [60]. To fill access gaps and offer assistance to persons in underserved locations, telehealth services and community outreach initiatives can be integrated [61]. In addition, policy advocacy is essential for addressing the structural causes of access gaps and promoting fair access to neurorehabilitation treatments [62]. These initiatives do not promote moral standards and contribute to lowering health inequalities and enhancing the well-being of underprivileged populations.

Integrating Neuroplasticity Insights Into Mainstream Clinical Practice

Although neuroplasticity has great promise, it is not easy to incorporate these understandings into therapeutic practice. Some practitioners may only partially understand the most recent findings on neuroplasticity and its implications. Continuous education and training are crucial for bridging the gap between research and practice [63]. Utilizing cutting-edge biomarkers and evaluation techniques is necessary to customize therapies to individual neuroplasticity responses. A problem that has to be solved is creating standardized techniques for evaluating neuroplasticity and responsiveness to therapies [2].

Careful management of time and resource is required for the incorporation of neuroplasticity discoveries into accepted clinical practice. Frequent and lengthy rehabilitation sessions are necessary for the best recovery with neuroplasticity-based rehabilitation, which sometimes necessitates a significant time commitment from patients and healthcare providers [64]. Providing resources for specialized training programs is required since ensuring healthcare personnel receive proper training is crucial for successfully implementing these measures [51]. Although cost-effectiveness and resource allocation must be considered, specialized facilities and technology may be essential for supporting neuroplasticity-focused therapies [25,50]. To successfully implement neuroplasticity-based rehabilitation, resource utilization must be optimized by considering patient motivation and active involvement [65].

Potential of Combined Therapies for Enhanced Neuroplasticity

Although there are difficulties, the prospect of integrating several neurorehabilitation therapies for improved neuroplasticity is encouraging. Although combining treatments may synergistically affect neuroplasticity, the best combinations and sequencing call for more research and clinical studies [66]. Individualization takes sophisticated prediction models and biomarkers to customize combination treatments to specific patient demands and responses. It is not easy to create personalized procedures for treatments that involve many therapies [39]. Coordination between various healthcare professionals is necessary to include multiple therapies in an extensive neurorehabilitation strategy. It is crucial to ensure efficient cooperation and communication among experts [67].

In conclusion, as neurorehabilitation develops, addressing ethical issues is crucial, incorporating neuroplasticity knowledge into practice, and maximizing the benefits of combination treatments. Overcoming these obstacles will open the door for more efficient and morally upright neurorehabilitation methods.

## Conclusions

The symbiotic link between neuroplasticity and recovery techniques predominates in the field of brain injury rehabilitation and provides a road map for post-injury restoration. The progression from damage to healing occurs against a background of complex neuroplastic alterations, highlighting the brain's inherent flexibility. Understanding cellular mechanics, synaptic plasticity, and adaptation sheds light on the brain's reaction to difficulty. The development of tactics that make use of the brain's built-in flexibility is fueled by this insight. Cognitive rehabilitation, VR, BCIs, and CIMT for focused healing use neuroplasticity. Yet, complexities emerge. To ensure that patient welfare remains the top priority, ethical concerns highlight the necessity for innovation within acceptable ethical bounds. It takes a transforming journey that connects theory with practical outcomes to integrate neuroplasticity insights into medical practice. Combination therapy's potential illuminates synergistic opportunities and amplifies neuroplastic effects. This highlights the dynamic environment of future neurorehabilitation and invites more research and collaboration. This review focuses on the crucial part neuroplasticity plays in brain damage healing. Neuroplasticity emerges as the beacon guiding the way ahead as research and practice unite, navigating the complex landscape of brain damage. Researchers and clinicians map pathways of recovery that improve the lives of persons affected by brain damage by utilizing the potential of neuroplasticity.
